# Heart failure with preserved ejection fraction shows no excess mortality in older patients: a population-matched relative survival analysis

**DOI:** 10.1007/s11357-025-02063-0

**Published:** 2026-01-06

**Authors:** Christophe de Terwangne, Nassiba Menghoum, Marin Boute, Sibille Lejeune, Agnès Pasquet, Bernhard Gerber, David Vancraeynest, Benoit Boland, Christophe Beauloye, Anne-Catherine Pouleur

**Affiliations:** 1 Division of Geriatric Medicine, Cliniques universitaires Saint-Luc – Université Catholique de Louvain, Brussels, Belgium; 2 Division of Cardiology, Cliniques universitaires Saint-Luc – Université Catholique de Louvain, Brussels, Belgium; 3 Pôle de Recherche Cardiovasculaire (CARD), Institut de Recherche Experimentale Et Clinique (IREC), Université Catholique de Louvain (UCLouvain), Brussels, Belgium; 4 Institut de Recherche Santé Et Société (IRSS), Université Catholique de Louvain, Brussels, Belgium; 5 Division of Intensive Care, Cliniques universitaires Saint-Luc – Université Catholique de Louvain, Brussels, Belgium

**Keywords:** Heart failure, Preserved ejection fraction, Diastolic, Survival, Older, Adjusted survival

## Abstract

Both heart failure with preserved ejection fraction (HFpEF) and advanced age are associated with increased mortality. However, HFpEF patients have not been compared with a similar age group from the general population. This study aimed to compare the mortality of HFpEF patients with an age- and gender-matched population, for two age groups (< 80 years and ≥ 80 years). Real-life cohort study of HFpEF patients with complete cardiac assessments carried out at an academic hospital since 2015. The observed mortality rates of the two age groups were compared to the expected mortality rate, using standardised mortality ratios (SMR). Of 334 HFpEF patients (mean age 79 ± 8.2 years, 64% women), 166 (50%) were ≥ 80 years. Older patients ≥ 80 years, compared to younger ones, had more frequently atrial fibrillation (73% vs. 61%), chronic kidney disease (67% vs. 53%), but less diabetes (24% vs. 45%), and lower BMI. During the follow-up (median: 4.1 years), 108 patients (32%) died. Compared to an age- and gender- matched population, HFpEF patients < 80 years had a three times higher mortality risk (SMR 3.37, 95% CI: 2.4–4.7), while HFpEF patients ≥ 80 years had a similar mortality risk (SMR 1.13, 95% CI:0.84–1.15). Consistently, no excess mortality was observed in the older patient group. Excess mortality is observed in HFpEF patients < 80 years but not in patients ≥ 80 years. This unexpected divergence likely reflects distinct HFpEF phenotypes and demographics across age groups. This trial was registered on 4 December 2014 at ClinicalTrials.gov (NCT03197350).

**Graphical Abstract:**

**Figure:**
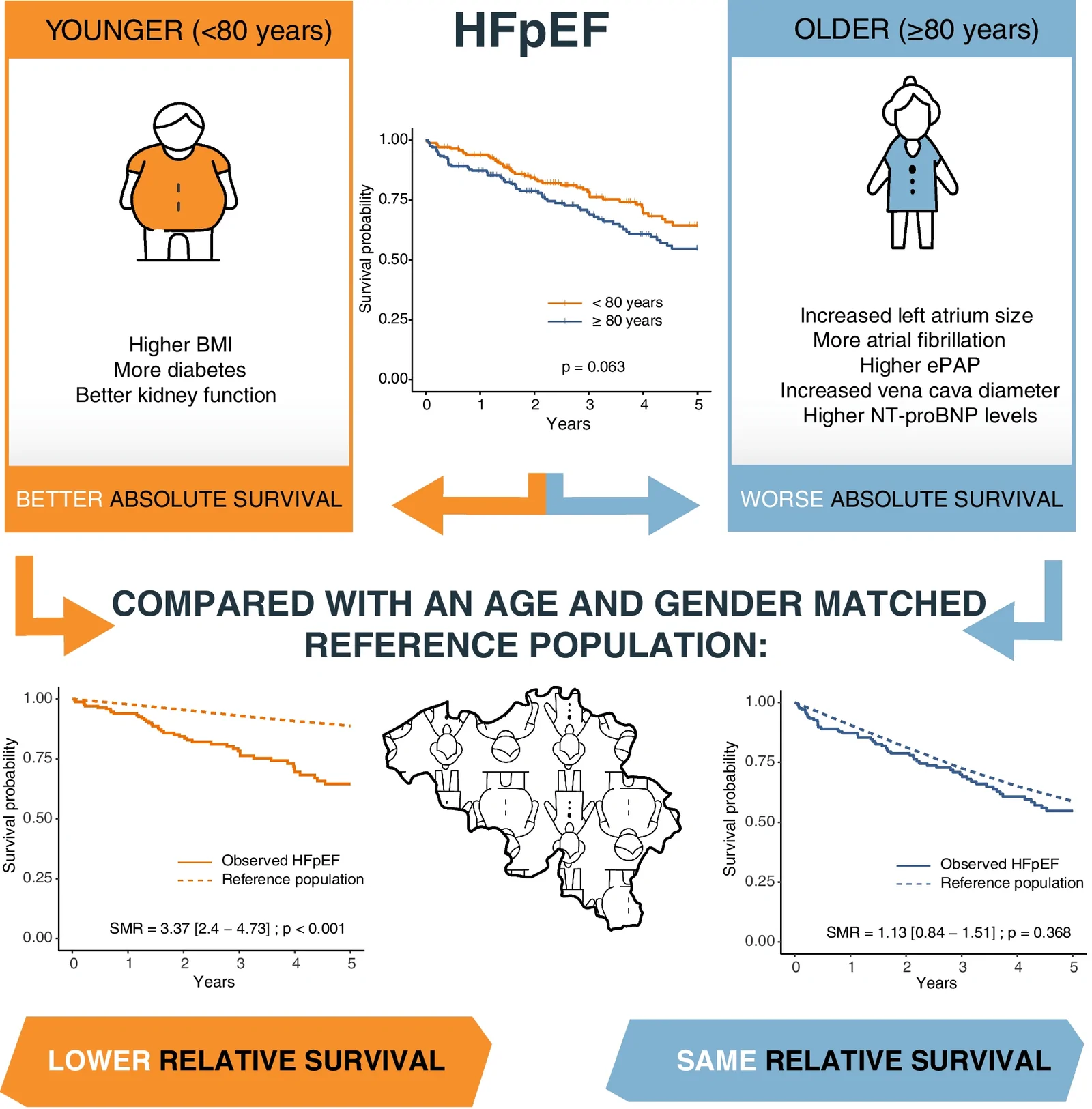

**Supplementary Information:**

The online version contains supplementary material available at https://doi.org/10.1007/s11357-025-02063-0.

## Background

Heart failure with preserved ejection fraction (HFpEF) is a poorly understood syndrome that disproportionately affects older patients. Different physiopathological mechanisms have been proposed to explain the close relationship between HFpEF and age, including age-related cardiovascular (CV) modifications and comorbidity accumulation. The higher comorbidity prevalence in HFpEF compared with heart failure (HF) with reduced ejection fraction has led to the hypothesis that they play a causal role in the development of the disease [1]. These conditions, e.g., arterial hypertension, obesity, diabetes, and chronic kidney failure, have been associated with the complex pathogenesis of various structural heart remodelling pathways, resulting in left chamber hypertrophy, stiffness, and myocardial fibrosis via a systemic pro-inflammatory state [2, 3]. This remodelling process eventually leads to impaired diastolic function and may result in the clinical expression of HF [4]. The wide range of different life exposures and trajectories of comorbid conditions inherently results in a high degree of age-related heterogeneity in patients evolving to HFpEF, to the extent that HFpEF in younger and older patients might differ in phenotype [5]. Furthermore, a previous report with pooled patients from different randomised controlled trials pointed out that older patients (> 85 years) had higher rates of all-cause mortality, CV mortality, non-CV mortality, and hospitalisations for HF [6]. Similarly, a Japanese cohort showed an incremental trend in all-cause mortality rates with advancing age [7].

However, to compare stratified mortality risks by age class, adjustment with an age- and gender-matched reference population is essential for an accurate and meaningful analysis of patient outcomes [8]. An index often used for this purpose is the ratio between the observed and expected death numbers of a standard reference population, called the standardised mortality ratio (SMR) [9, 10].

In such relative survival frameworks, excess mortality (sometimes called cause-specific mortality) refers to mortality among patients with the disease that exceeds the mortality expected from age–sex–calendar-year–matched general-population life tables [11].

Therefore, the present study sought 1) to compare survival of older (≥ 80 years) and younger (< 80 years) real-life Belgian HFpEF patients during a 5-year follow-up, and 2) to compare the observed survival of both age groups with the expected survival of an age- and gender-matched reference population using a relative survival framework. Additionally, the characteristics of older (≥ 80 years) and younger (< 80 years) real-life Belgian patients with HFpEF were compared.

## Methods

### Study design and patients

This cohort study was conducted at the Cliniques universitaires Saint-Luc, a tertiary hospital centre in Brussels, Belgium. HFpEF patients were prospectively enrolled from the outpatient and inpatient clinics of the cardiology department between 2015 and 2023. The complete study design description has been previously reported [12]. Briefly, patients were eligible for enrolment if they fulfilled the following criteria: New York Heart Association (NYHA) functional Class ≥ II, typical HF signs, NT-proBNP level > 350 pg/mL and/or hospitalisation for HF in the previous 12 months, LVEF ≥ 50%, and relevant structural heart disease (i.e., diastolic dysfunction according to the American Society of Echocardiography criteria) based on echocardiography [12, 13]. Patients were excluded if they presented a history of reduced ejection fraction (LVEF < 50%) or one of the following diseases: severe valvular disease, infiltrative or hypertrophic cardiomyopathy, acute coronary syndrome in the previous 30 days, severe chronic obstructive pulmonary disease, congenital heart disease, pericardial disease, AF with a ventricular response > 140 bpm at inclusion, and severe anaemia (haemoglobin < 8 g/dL). Patients’ characteristics, treatments, as well as general and CV history were collected at enrolment along with blood sampling and full transthoracic echocardiography. Hypercholesterolaemia was defined as either a total cholesterol level exceeding the European Cardiology Society guidelines or statin use for this indication. History of atherosclerotic disease included history of peripheral artery disease, percutaneous transluminal coronary angioplasty, coronary artery bypass surgery, and neurovascular disease.

All participants underwent a standardised transthoracic echocardiography, using an iE33 system Philips Healthcare Best (The Netherlands), following the American Society of Echocardiography recommendations to assess left and right ventricular structure, systolic and diastolic function, and measurements of left and right atrial volumes[14]. All measures were averaged over three beats in case of AF. Estimated pulmonary artery pressure (ePAP) was obtained by adding to the tricuspid valve gradient either 5 mmHg or 15 mmHg in cases where the inferior vena cava diameter was below or above 21 mm, respectively.

Patients were prospectively followed up every 6 months by ambulatory visits or phone calls. The primary endpoint was all-cause mortality.

### Statistical analysis

Statistical analysis was performed using R (version 4.3.2, R Core Team, Vienna, Austria, 2022). Categorical variables were expressed as counts and percentages. Continuous variables were reported as means (± standard deviations [SDs]) or medians [25th–75th percentiles], as appropriate. The cohort was divided into age groups: younger patients were < 80 years old and older patients were ≥ 80 years old.

*Age-related characteristics*: Differences in characteristics between age groups were assessed using the χ^2^ test or Fisher’s exact test for categorical variables and the Wilcoxon Rank-sum test for continuous variables. Bivariable logistic regression was used to study associations between characteristics and age groups. Bivariable Cox regression analysis was used to assess which characteristics were predictive of mortality among age groups.

*Survival analysis*: Overall survival curves were estimated by the Kaplan-Meier method and a two-sample log-rank test was used to assess the difference in survival between age groups. To compare the observed survival of each age group to that of an age- and gender-matched reference population (general Belgian population), a relative survival ratio (the SMR) was computed according to Finkelstein et al. [15]. Briefly, the annual death rates for each year, age, and gender were retrieved from standardised national Belgian actuary tables [16]. The annual death rate was used to compute the expected cumulative death rate for each subject, matched for the subject’s age and gender, each year after enrolment. The cumulative death rate at each subject’s time of censoring or death allowed the calculation of the total expected death number at the end of follow-up (5 years). Then, the SMR was computed by expressing the ratio between observed and expected death numbers at 5 years. A one-sample log-rank test was subsequently performed to assess the difference in the observed and expected death numbers in each age group [15]. A complementary analysis was performed to assess SMR consistency for both age groups at different censored follow-up times (1 year, 3 years, and 5 years). Only for this purpose, age classes of 5-year intervals were created.

Using the relsurv package (i.e., Pohar-Perme methodology), excess mortality was computed for each age strata (< 80 and ≥ 80 years old) [17]. To explore the effect of comorbidity burden on excess mortality in each age stratum, the amount of major comorbidities (hypertension, atrial fibrillation, chronic kidney disease with eGFR < 30 ml/min, cardiac or peripheral atherosclerotic disease, chronic obstructive pulmonary disease, anaemia, obstructive sleep apnea and prior stroke; 1 point per condition; range 0–8) was included in multiplicative relative survival models along with age category and their interaction.

Throughout the analysis, a p-value < 0.05 was considered statistically significant, and a p-value between 0.05 and 0.1 was considered a statistical trend.

## Results

### Patient characteristics

**Fig. 1:**
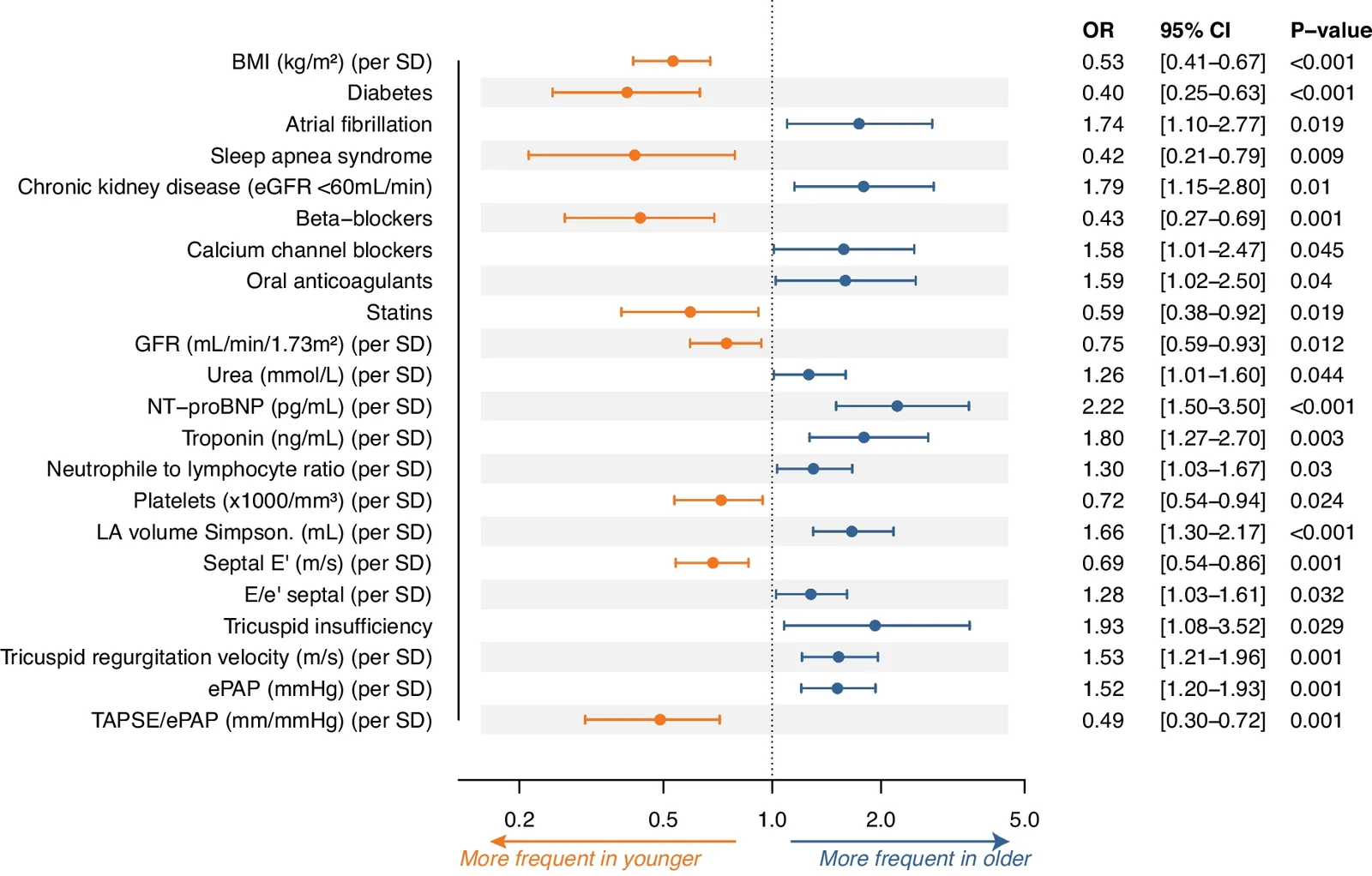
Significant (*p* < 0.05) differences regarding baseline characteristics between older (≥ 80 years) and younger (< 80 years) HFpEF patients. Bivariable logistic regression analysis. BMI: body mass index; CI: confidence interval; eGFR: estimated glomerular filtration rate; ePAP: estimated pulmonary artery systolic pressure; HFpEF: heart failure with preserved ejection fraction; LA: left atrial; NT-proBNP: N-terminal pro b-type natriuretic peptide; OR: odds ratio; SD: standard deviation; TAPSE: tricuspid annular plane systolic excursion

Of the 334 included patients (median age: 79 [73–85] years, 64% women), 166 (50%) were ≥ 80 years old. The median age of the older group was 85 [83–88] years, whereas that of the younger group was 73 [69–77] years old. Comparisons of baseline characteristics between age groups are presented in Table 1. Compared with the younger patients, older patients were more likely to have AF (odds ratio [OR]: 1.74, 95% confidence interval [CI] [1.10–2.77]) and chronic renal failure (OR: 1.79, 95% CI [1.15–2.80]). They presented higher NT-proBNP values (OR: 2.22 per SD, 95% CI [1.50–3.50]), troponin levels (OR: 1.80 per SD, 95% CI [1.27–2.70]), urea levels (OR: 1.26 per SD, 95% CI [1.01–1.60]), indexed left atrial volume (OR: 1.66 per SD, 95% CI [1.30–2.17]), and ePAP (OR: 1.43 per SD, 95% CI [1.15–1.81]) (Fig. 1). The association between older age and both NT-proBNP and troponin levels remained significant (p < 0.01) after adjusting for BMI, creatinine level, and gender (Table S1). Older patients, compared with younger patients, were less likely to have diabetes (OR: 0.40, 95% CI: 0.25 to 0.63). They had lower BMI (OR: 0.53 per SD, 95% CI [0.41–0.67]), estimated glomerular filtration rate (eGFR) (OR: 0.75 per SD, 95% CI [0.59–0.93]), tricuspid annular plane systolic excursion (TAPSE)/ePAP ratios (OR: 0.41 per SD, 95% CI [0.17–0.73]), and septal E’ velocity (OR: 0.69 per SD, 95% CI [0.54–0.86]) (Fig. 1.).

**Table 1:** Clinical characteristics of patients by age group (< 80 years vs. ≥ 80 years)

	Overall N = 334^*1*^	< 80 years N = 168^*1*^	≥ 80 years N = 166^*1*^	p-value^*2*^
DEMOGRAPHICS				
Age (years)	79 (73, 85)	73 (69, 77)	85 (83, 88)	** < 0.001**
Gender (male)	120 (36%)	61 (36%)	59 (36%)	0.9
BMI (kg/m^2^)	28 (24, 33)	30 (25, 36)	26 (23, 29)	** < 0.001**
NYHA Class 3–4	131 (39%)	65 (39%)	66 (40%)	0.8
H2FPEF score	6 (5, 7)	6 (5, 8)	7 (5, 7)	0.9
CARDIOVASCULAR RISK FACTORS				
Smoking	119 (36%)	65 (39%)	54 (33%)	0.3
Hypertension	313 (94%)	155 (92%)	158 (96%)	0.2
Diabetes	115 (35%)	75 (45%)	40 (24%)	** < 0.001**
Family history of CAD	73 (22%)	43 (26%)	30 (18%)	0.10
Hypercholesterolaemia	224 (67%)	114 (68%)	110 (67%)	0.8
MEDICAL HISTORY				
Atrial fibrillation	223 (67%)	102 (61%)	121 (73%)	**0.018**
Previous HF episodes	211 (63%)	109 (65%)	102 (61%)	0.5
Previous valvular surgery	39 (12%)	18 (11%)	21 (13%)	0.6
Atherosclerotic disease	168 (50%)	78 (46%)	90 (54%)	0.2
Permanent pacemaker	38 (11%)	15 (8.9%)	23 (14%)	0.2
Chronic obstructive pulmonary disease	30 (9.0%)	15 (8.9%)	15 (9.0%)	> 0.9
Sleep apnoea syndrome	48 (15%)	33 (20%)	15 (9.3%)	**0.007**
Chronic renal disease (eGFR < 60 mL/min)	200 (60%)	89 (53%)	111 (67%)	**0.010**
MEDICATION				
Diuretics	258 (77%)	129 (77%)	129 (78%)	0.8
Spironolactone	80 (24%)	43 (26%)	37 (22%)	0.5
Beta-blockers	229 (69%)	130 (77%)	99 (60%)	** < 0.001**
ACE inhibitors/ARB	224 (67%)	113 (67%)	111 (67%)	> 0.9
Calcium channel blockers	125 (37%)	54 (32%)	71 (43%)	**0.045**
Antiplatelet agent	88 (26%)	44 (26%)	44 (27%)	> 0.9
Oral anticoagulation	207 (62%)	95 (57%)	112 (67%)	**0.040**
Statins	170 (51%)	97 (58%)	73 (45%)	**0.018**
LABORATORY				
CRP (mg/L)	1.4 (0.6, 4.6)	1.3 (0.6, 4.6)	1.6 (0.6, 4.7)	0.5
Creatinine (mg/dL)	1.12 (0.90, 1.45)	1.11 (0.88, 1.42)	1.14 (0.90, 1.53)	0.3
eGFR CKD-EPI (mL/min/1.73m^2^)	52 (38, 66)	55 (41, 70)	49 (37, 63)	**0.004**
Urea (mmol/L)	51 (39, 73)	49 (38, 67)	54 (40, 76)	**0.048**
Sodium (mmol/L)	139 (137, 141)	139.0 (137, 141)	140.0 (137, 141)	0.6
Haemoglobin (g/dL)	12.3 (10.8, 13.4)	12.4 (11.1, 13.5)	12.3 (10.7, 13.4)	0.4
NT-proBNP (pg/mL)	1,460 (558, 3,182)	1,158 (455, 2,402)	1,925 (1,025, 4,226)	** < 0.001**
Troponin (ng/mL)	22 (13, 37)	18 (11, 31)	30 (17, 42)	** < 0.001**
Total cholesterol (mmol/L)	156 (130, 184)	152 (133, 180)	162 (129, 188)	0.7
LDL cholesterol (mg/dL)	77 (56, 97)	75 (54, 94)	81 (59, 100)	0.2
HDL cholesterol (mmol/L)	52 (42, 65)	51 (42, 63)	54 (41, 66)	0.9
Triglycerides (mmol/L)	101 (76, 135)	107 (81, 139)	95 (72, 132)	0.11
Neutrophils (× 1,000/mm^3^)	4.69 (3.62, 6.06)	4.74 (3.64, 6.10)	4.58 (3.62, 6.06)	0.6
Lymphocytes (× 1,000/mm^3^)	1.47 (1.07, 1.94)	1.60 (1.19, 2.04)	1.33 (1.03, 1.78)	**0.003**
Platelets (× 1,000/mm^3^)	228 (185, 285)	236 (195, 291)	219 (177, 262)	**0.004**
ECHOCARDIOGRAPHY				
LA volume Simpsonᵢ (mL)	40 (33, 50)	37 (29, 46)	43 (35, 54)	** < 0.001**
LVEDV Simpsonᵢ (mL)	57 (47, 69)	57 (49, 68)	57 (46, 69)	0.7
LVESV Simpsonᵢ (mL)	22 (17, 28)	22 (18, 28)	21 (17, 29)	0.5
LVEF Simpsonᵢ	60 (55, 66)	60 (56, 65)	60 (55, 66)	0.5
SV (mL)	58 (46, 72)	58 (48, 75)	57 (45, 70)	0.2
Mitral E (cm/s)	95 (76, 116)	95 (77, 118)	93 (75, 115)	0.6
Septal E' (m/s)	5.63 (4.60, 6.78)	5.82 (4.90, 7.20)	5.30 (4.40, 6.40)	**0.001**
E/e' septal	16 (13, 21)	15 (13, 19)	17 (13, 23)	**0.040**
Tricuspid insufficiency ≥ 2	57 (17%)	21 (13%)	36 (22%)	**0.027**
RV FAC (%)	42 (35, 48)	41 (35, 48)	42 (34, 49)	0.5
TAPSE (mm)	19.0 (15.0, 23.0)	20.0 (14.7, 24.0)	18.0 (15.0, 21.0)	0.068
Tricuspid regurgitation velocity (m/s)	2.90 (2.50, 3.19)	2.77 (2.45, 3.11)	2.96 (2.63, 3.28)	**0.001**
ePAP (mmHg)	40 (31, 50)	37 (29, 46)	43 (34, 52)	** < 0.001**
TAPSE/ePAP (mm/mmHg)	0.56 (0.42, 0.82)	0.66 (0.44, 0.92)	0.54 (0.41, 0.68)	**0.002**

^*1*^Median (Q1, Q3); n (%)

^*2*^Wilcoxon rank sum test; Pearson's Chi-squared test

Abbreviations: *ACE* angiotensin-converting enzyme, *ARB* angiotensin receptor blocker, *BMI* body mass index, *CAD* coronary artery disease, *CKD-EPI* chronic kidney disease—epidemiology collaboration, *CRP* C-reactive protein, *eGFR* estimated glomerular rate, *ePAP* estimated pulmonary artery systolic pressure, *HDL* hidh-density lipoprotein, *HF* heart failure, *LA* left atrial, *LDL* low-density lipoprotein, *LVEDV* left ventricular end‐diastolic chamber volume, *LVEF* left ventricular ejection fraction, *LVESV* left ventricular end‐systolic chamber volume, *NO* nitric oxide, *NT-proBNP* N-terminal pro b-type natriuretic peptide, *NYHA* New York heart association, *RV FAC* right ventricular fractional area change, *SV* stroke volume, *TAPSE* tricuspid annular plane systolic excursion

### Mortality

In the bivariable survival analysis, mortality hazard increased in both younger and older patients with chronic kidney disease (CKD), higher NT-proBNP values, higher troponin levels, and higher ePAP (Table S2), while higher haemoglobin levels, eGFR, high density lipoprotein (HDL)-cholesterol levels were protective.

However, the two age groups differed regarding several factors associated with mortality hazard (Table S2). Indeed, among younger patients, previous acute HF episode, treatment with antiplatelet agents, and higher C-reactive protein levels were found to be predictive of mortality (HR > 1, p < 0.05). By contrast, in these younger patients, being female, having a higher BMI, and having better right ventricular function were protective (HR < 1, p < 0.05). In older patients, diabetes, valvular surgery history, higher white blood cell count, and worse diastolic function (left atrial volume and E/e’) increased the death risk (HR > 1, p < 0.05), whereas treatment with statins decreased it (HR < 1, p < 0.05).

### Survival

**Fig. 2:**
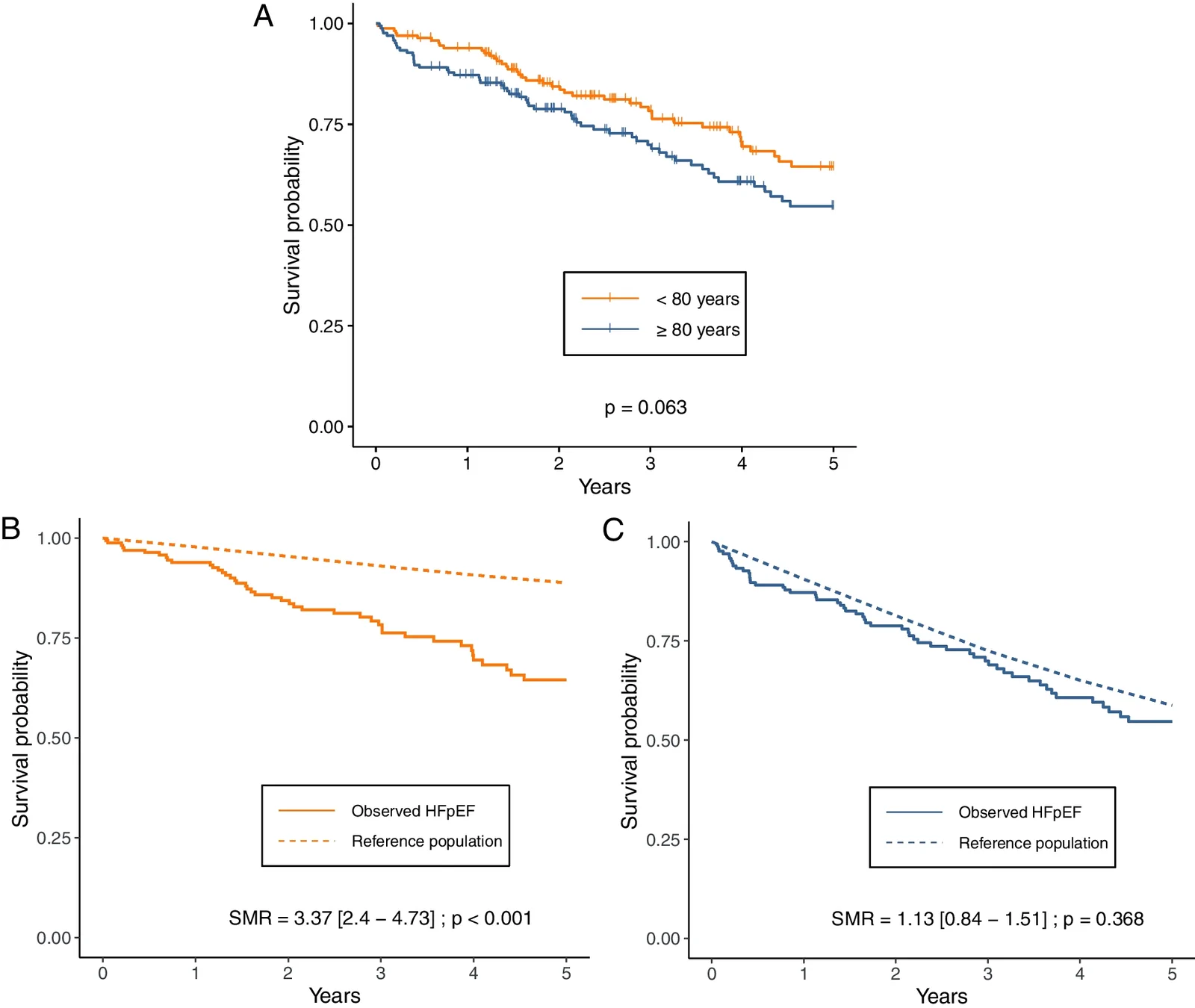
**A**) Kaplan Meier survival curve of HFpEF patients stratified by age groups (orange < 80 years, blue ≥ 80 years), **B**) observed survival of patients aged < 80 years compared with an age- and gender-matched reference population, and **C**) observed survival of patients aged ≥ 80 years compared with an age- and gender-matched reference population. SMR = standardised mortality ratio at 5 years. HFpEF: heart failure with preserved ejection fraction

During a median follow-up of 4.12 [3.73–5.14] years, 108 (32%) patients (47 younger and 61 older patients) died. Older patients showed a non-significantly lower survival rate compared with younger patients (log-rank p = 0.063) (Fig. 2A). The mortality hazard as a function of age showed a marked increase in risk after the age of 80 (Figure S1).

**Fig. 3:**
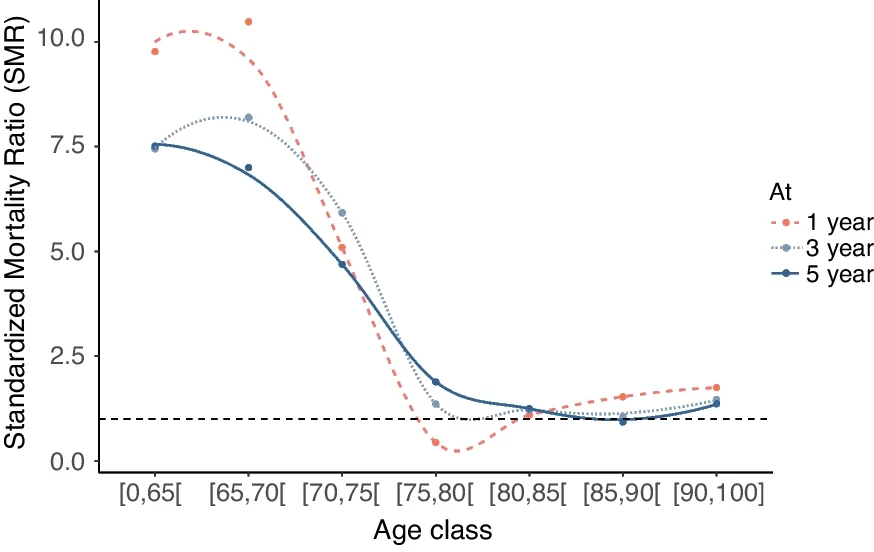
Standardised mortality ratios (SMRs) per 5-year age class at 1 year, 3 years, and 5 years of follow-up. The dashed line represents SMR = 1

An SMR of 1.58 (95% CI [1.26–1.97]) was computed for the entire study group (n = 334), with a significant difference between observed and expected mortality rates (p < 0.001). However, when compared to their age- and gender-matched population, younger HFpEF patients had a significantly three times higher mortality rate (SMR 3.37, 95% CI [2.4–4.7], p < 0.001) (Fig. 2B), whereas older HFpEF patients had the same mortality rate (SMR 1.13, 95% CI [0.84–1.15], p = 0.368) as their reference population (Fig. 2C). SMR evolution for the 5-year age classes (Fig. 3) showed a marked increase in mortality risk, as compared to the age- and gender-matched population, for all age classes before 75–80 years old. By contrast, the SMR was comparable with that of the matched reference population for all older age classes. The difference was observed across different censored follow-up times, i.e., 1, 3, and 5 years (Fig. 3).

**Fig. 4:**
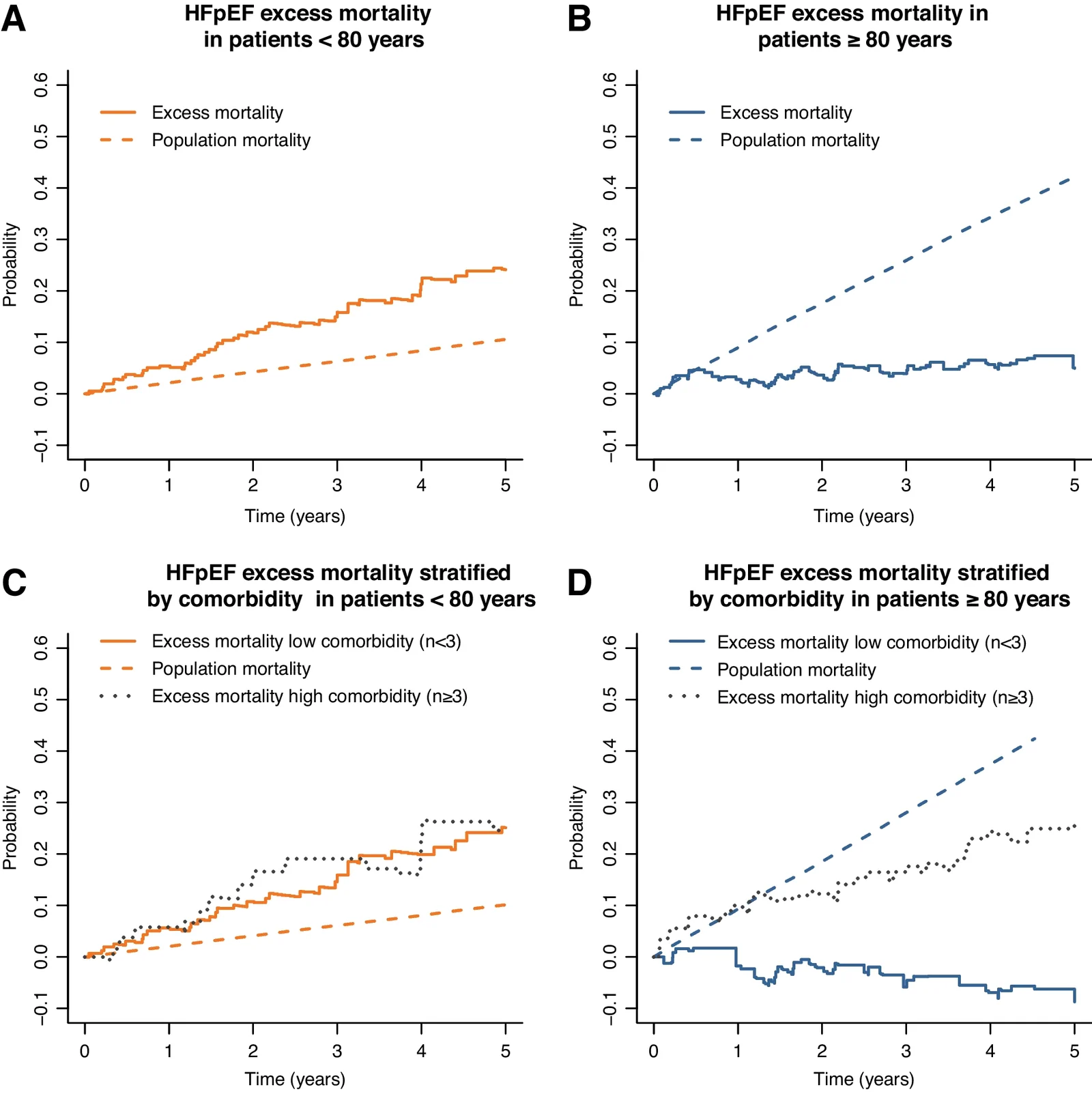
HFpEF excess mortality compared to the reference population in younger patients (< 80 years) (**A**) and older patients (≥ 80 years) (**B**). Excess HFpEF mortality stratified by low and high comorbidity in younger patients (**C**) and older patients (**D**)

Excess mortality due to HFpEF over time was also computed, as illustrated in Fig. 4. While the probability of excess mortality in older patients stayed below 0.1 over time (Fig. 4B), it progressively increased in younger patients (Fig. 4A).

A significant interaction between age and comorbidity burden (multiplicative relative survival model, interaction term HR 1.43 per additional comorbidity, 95% CI 1.01–2.01, p = 0.048) has been observed, suggesting that comorbidity burden increases excess mortality in patients ≥ 80 years, whereas its effect on excess mortality in those < 80 years remains small. The stratified relative survival curves (Fig. 4C) show that, in younger HFpEF patients, the excess risk over time is similar regardless of comorbidity burden. In contrast, among older patients (Fig. 4D), a high comorbidity burden (n ≥ 3) substantially raises excess mortality, while those with fewer comorbidities experience slightly negative excess mortality. This suggests that “fit” patients with HFpEF experience less mortality compared to the standard population. This has to be interpreted cautiously, as it likely reflects the selection of particularly “fit” older individuals rather than a true protective effect of HFpEF.

## Discussion

This real-life study of Belgian HFpEF patients highlighted interesting findings. Firstly, the clinical characteristics of older HFpEF patients largely differed from those of younger HFpEF patients. Secondly, in this cohort, the absolute survival difference between age groups was moderate, but presented a marked increase in the absolute mortality risk starting at approximately 80 years old. Thirdly, in contrast with the previous finding, the relative mortality risk of older HFpEF patients was similar to that of their matched reference population, whereas younger HFpEF patients had a significantly three-fold higher relative mortality risk compared with their matched reference population. In line with the latter, older patients with HFpEF present no excess mortality compared to age‑, sex‑ and calendar year‑matched general population.

### Age-related clinical profiles and physiopathological mechanisms

Tromp et al. pooled 8,468 HFpEF patients with a left ventricular ejection fraction (LVEF) ≥ 45% from the TOPCAT, PRESERVE, and CHARM trials. They found that younger patients (< 65 years old) were more likely to be obese men with a lower comorbidity burden, whereas older HFpEF patients were more often white women with a higher comorbidity burden [6]. A more recent Japanese study selected 2,824 patients from the CHART-2 cohort study with stage C/D chronic HF with LVEF ≥ 50%. They pointed out that younger HFpEF patients (< 65 years old) were predominantly males with an elevated body mass index (BMI) and poorly controlled diabetes, whereas older HFpEF patients were more likely to be females with multiple comorbidities such as coronary artery disease (CAD), hypertension, renal impairment, and atrial fibrillation (AF) [7].

In line with those previous studies, younger HFpEF patients (< 80 years) in our study also exhibited a distinct profile characterised by higher BMI, diabetes prevalence, obstructive sleep apnoea, and more frequent use of statins. This clustering of seemingly metabolic risk factors in younger HFpEF patients indicated a higher prevalence of metabolic syndrome in those patients, which is increasingly recognised as a significant HFpEF precursor [18]. By contrast, older HFpEF patients presented with different clinical characteristics, including AF and CKD incidence, lower BMI, larger left atrial volumes, as well as higher E/e’ and ePAP. These features are more in line with the CV changes generally seen in the ageing CV system, as well as a previously identified ageing HFpEF phenotype [19]. Indeed, as individuals age, structural and functional changes in the micro and macro CV system, such as increased arterial stiffness, left ventricular hypertrophy, and atrial enlargement, lead to increased vascular resistance and reduced compliance of the large arteries, myocardium, and atrium, which are hallmarks of diastolic dysfunction, atrial myopathy, and ultimately clinical HF [20, 21]. The differences in profiles between younger and older HFpEF patients imply that structural changes leading to HFpEF may result from different mechanisms, even though common physiological pathways between metabolic and age-related CV changes may occur [6, 7, 22]. These results also support findings from the bivariable analyses that identified different mortality predictors in younger and older HFpEF patients, respectively.

### Survival

The present analysis showed increased absolute all-cause mortality in older HFpEF patients compared with younger ones (Fig. 2A, Figure S1). However, by comparing mortality figures of the present cohort with that of a matched reference population, our data suggested a three times higher relative mortality risk in younger HFpEF patients compared with their matched population (SMR = 3.37). By contrast, a relative mortality risk equivalent to that of their matched population (SMR = 1.13) was reported in older HFpEF patients. Of note, the SMR of 1.58 of our entire population (mean age: 79 years) was comparable to that of a previous study that assessed expected and observed mortality rates of HFpEF patients (mean age: 76 years) and computed a relative survival at 5 years of 60% (corresponding to an SMR of 2.03) [23]. However, the latter study did not stratify relative rates by age.

When cause of death is unknown or unreliable, a relative survival framework is useful to estimate excess mortality hazard attributable to the disease, under the assumption that this expected hazard approximates what patients would experience in the absence of the disease (HFpEF in this study) [17]. Within this framework, the hazard attributable to HFpEF (λ_HFpEF_), or excess mortality, corresponds to the overall hazard (λ_O_) minus the hazard of dying from other causes (λpop), known as the population hazard [24]. The population hazard (λpop) is computed from the mortality rates retrieved in the national actuary tables after age-, sex-, and calendar year-matching with the observed patients sample.

In contrast, the age-stratified Kaplan Meier curve (Fig. 2A) represents the overall survival function for each age strata, directly related to the overall mortality hazard (λ_O_), and does therefore not distinguish the hazard of mortality due to the disease, or excess mortality (λ_HFpEF_), and the hazard of dying from other causes (λpop). Age-stratified Kaplan–Meier survival curves should therefore be interpreted very cautiously.

In that respect, using relative survival analysis, we showed that while the population mortality hazard was increased in the older group, the HFpEF excess mortality was lower in older HFpEF patients than in younger patients (Fig. 4). This striking difference may be supported by different hypotheses.

First, former studies have revealed distinct HFpEF phenotypes associated with metabolic, overweight, and inflammatory profiles. Higher comorbidity prevalence and poorer health status typically characterise these phenotypes. Notably, patients with these phenotypes tend to be younger and experience worse prognoses compared with non-metabolic phenotypes [25–28]. In a relative survival construct, this could be translated into a more pronounced difference in health, and by extension, mortality hazard, between those with HFpEF and their matched reference in the younger stratum. The latter could inflate excess mortality due to HFpEF in this stratum. While metabolic risk profiles may explain poor outcomes in younger patients, the relatively preserved prognosis of older HFpEF patients may stem from different demographic and biological considerations.

Underlying processes of ageing lead to an increase of all-cause mortality at a set pace with every year as we become older [29]. During adulthood, the mortality risk increases exponentially by about 11% between successive ages [30, 31]. The rising mortality risk is commonly referred to as the rate-of-ageing and has been plotted for the Belgian population (Figure S2). In this study, the mortality hazard of the reference population (λpop) is largely driven by the rate of aging, which reflects the progressive accumulation of comorbidity and frailty with advancing age. When the expected mortality rate is already high in the matched population, an additional effect from HFpEF may reach a ceiling, making the relative increase in hazard appear smaller.

Another hypothesis is that patients ≥ 80 years who survive HFpEF may represent a selected subgroup with greater biological resilience and lower vulnerability to HF-related complications. This survivor effect could also attenuate the estimated excess mortality in the oldest age stratum.

While life tables do not contain information on frailty or comorbidity at the individual level, the relative survival analysis reflects the aggregate burden of comorbidities and ageing-related conditions in the general population for a given age and sex. To overcome this limitation, multiplicative relative survival models were computed using the count of significant morbidities. These analyses illustrate the importance of the high heterogeneity in the older population and the significant prognostic impact of comorbidity in the oldest strata.

The important discrepancy between absolute and relative mortality risks necessitates a deeper understanding of the underlying mechanisms that might underscore important therapeutic targets between age groups. Currently, there is new hope with the development of sodium-glucose co-transporter-2 inhibitors. Moreover, other important efforts are being made to elaborate new pharmacological strategies to enhance survival in HFpEF patients [32, 33]. Our analysis indicated that older patients with HFpEF had worse absolute survival rates, but their HFpEF-driven excess mortality risk was comparatively lower. This finding also supports earlier research indicating that diastolic dysfunction, a common precursor to HFpEF in older people, has limited predictive value for mortality [34]. The diastolic dysfunction–HFpEF spectrum might therefore represent, to some extent, a compensatory response to the physiologic ageing of the cardiovascular system in this population.

This suggests that, in addition to optimal HF treatment, more comprehensive management strategies are essential to improve outcomes for older patients. Frailty, a common geriatric syndrome in this population, increases vulnerability to stressors and exacerbates HF symptoms, leading to a higher risk of adverse outcomes [35].

Targeting frailty and other geriatric syndromes might encourage a holistic patient-centred approach, helping clinicians align both pharmacological and non-pharmacological therapeutic strategies with the unique needs of older HFpEF adults, ultimately improving both survival and quality of life. Today, the complexity associated with (older) HFpEF patients represents one of the main challenges regarding treatment. Research into interdisciplinary care should, therefore, be encouraged.

### Strengths and limitations

This study is the first to use a relative survival framework to understand the prognosis of older HFpEF patients. This framework assumes that the population hazard approximates what our patients would experience in the absence of HFpEF. Altough, it must be noted that HFpEF prevalence has been suggested to increase with age and might thus impact our analysis by skewing the reference population [36]. The precise HFpEF prevalence, particularly in older patients, remains poorly described. A community-based study in Portugal (EPICA study) estimated the HFpEF prevalence in older patients (≥ 80 years) at 10% and 4% in females and males, respectively, while a Swedish population-based registry estimated the HFpEF prevalence at approximately 30% in people older than 85 years [37, 38].

Additionally, this study lacks frailty measures, which are increasingly recognised as closely associated with HF and worse prognosis, especially in the older population.

Given that the study cohort was drawn from a single geographic area, the generalisability of the findings to other populations may be limited. The current analysis complements earlier data from randomised controlled trials, however, by offering a real-world perspective.

Despite these limitations, the finding that HFpEF drives only limited excess mortality in older patients provides insights into its overall mortality impact in this group. These patients, often frail and with comorbidities, are more likely to die from other causes before HFpEF-related death. Future research should focus on disentangling the effects of various covariates, which could guide more tailored patient-centred care interventions for specific subgroups.

## Conclusion

In this study, HFpEF had an important prognostic impact in younger (< 80 years) patients but not in older patients. Differences in characteristics and prognosis between younger and older HFpEF patients indicate varying phenotypes with specific demographics, highlighting the need to shift treatment strategies in the older population from solely survival-focused to strategies emphasising functionality, quality of life, and personalised care.

## Supplementary Information

Below is the link to the electronic supplementary material.

Supplementary file1 (PDF 401 KB)

## Data Availability

The datasets used and/or analysed during the current study are available from the corresponding author on reasonable request.

## Abbreviations

AF: Atrial fibrillation
BMI: Body mass index
CKD: Chronic kidney disease
CAD: Coronary artery disease
CI: Confidence interval
CV: Cardiovascular
eGFR: Estimated glomerular filtration rate
ePAP: Estimated pulmonary artery systolic pressure
HF: Heart failure
HDL: High density lipoprotein
HR and aHR: (Adjusted) Hazard ratio
HFpEF: Heart failure with preserved ejection fraction
LVEF: Left ventricular ejection fraction
NYHA: New York heart association
OR: Odds ratio
SMR: Standardised mortality ratio
SD: Standard deviation
TAPSE: Tricuspid annular plane systolic excursion
