# Supplementary material for: Heart failure with preserved ejection fraction shows no excess mortality in older patients: a population-matched relative survival analysis
Source: GeroScience. 2026 Jan 6;48(6):7721–34. doi: 10.1007/s11357-025-02063-0 (PMC13638804; doi:10.1007/s11357-025-02063-0)
Supplement: Supplementary file 1 [file 11357_2025_2063_MOESM1_ESM.docx]

**APPENDICES**

|  | Table S1. Multivariable adjusted association between NT-proBNP and troponin levels with older age (dependent variable). | | | | | | | |
| --- | --- | --- | --- | --- | --- | --- | --- | --- |
|  | |  | | |  |  | | |
|  |  | **OR***^1^* | **95% CI***^1^* | **p-value** |  | **OR***^1^* | **95% CI***^1^* | **p-value** |
| **NT-proBNP (per SD)** | | 1.93 | 1.29, 3.13 | **0.004** | **Troponin (per SD)** | 1.84 | 1.23, 3.02 | **0.008** |
| Creatinine (mg/dL) (per SD) | | 1.04 | 0.78, 1.39 | 0.8 | Creatinine (mg/dL) (per SD) | 0.97 | 0.70, 1.35 | 0.9 |
| BMI (kg/m^2^) (per SD) | | 0.59 | 0.45, 0.75 | <0.001 | BMI (kg/m^2^) (per SD) | 0.57 | 0.43, 0.73 | <0.001 |
| Gender | |  |  |  | Gender |  |  |  |
| Man | | — | — |  | Man | — | — |  |
| Woman | | 1.22 | 0.73, 2.06 | 0.4 | Woman | 1.11 | 0.65, 1.91 | 0.7 |
|  | *^1^* BMI: body mass index; OR: odds ratio; CI: confidence interval; NT-proBNP: N-terminal pro b-type natriuretic peptide; SD: standard deviation. | | | | | | | |

| **Table S2. Bivariable Cox regression analysis of clinical characteristics** | | | | | | | | | | |
| --- | --- | --- | --- | --- | --- | --- | --- | --- | --- | --- |
|  | All | | | | Younger patient (<80 years old) | | | Older patient (≥80 years old) | | |
|  | **N** | **HR***^1^* | **95% CI***^1^* | **p-value** | **HR***^1^* | **95% CI***^1^* | **p-value** | **HR***^1^* | **95% CI***^1^* | **p-value** |
| Age (years) | 332 | 1.04 | 1.01, 1.06 | **0.004** | 1.00 | 0.96, 1.05 | 0.9 | 1.13 | 1.06, 1.21 | **<0.001** |
| Gender (Woman) | 332 | 0.65 | 0.44, 0.94 | **0.025** | 0.52 | 0.30, 0.93 | **0.028** | 0.78 | 0.46, 1.29 | 0.3 |
| BMI (kg/m²) (per SD) | 332 | 0.72 | 0.59, 0.88 | **0.001** | 0.61 | 0.44, 0.84 | **0.002** | 0.88 | 0.66, 1.17 | 0.4 |
| NYHA Class 3-4 | 332 | 1.17 | 0.80, 1.72 | 0.4 | 1.15 | 0.64, 2.05 | 0.6 | 1.18 | 0.71, 1.95 | 0.5 |
| Smoking | 331 | 1.08 | 0.73, 1.58 | 0.7 | 1.09 | 0.61, 1.95 | 0.8 | 1.10 | 0.65, 1.84 | 0.7 |
| Hypertension | 331 | 1.00 | 0.44, 2.28 | >0.9 | 0.88 | 0.31, 2.44 | 0.8 | 1.05 | 0.26, 4.32 | >0.9 |
| Diabetes | 331 | 1.37 | 0.93, 2.01 | 0.11 | 1.26 | 0.71, 2.23 | 0.4 | 1.83 | 1.08, 3.11 | **0.031** |
| Family history of CAD | 326 | 1.01 | 0.64, 1.61 | >0.9 | 0.75 | 0.37, 1.51 | 0.4 | 1.54 | 0.83, 2.84 | 0.2 |
| Hypercholesterolaemia | 331 | 0.75 | 0.51, 1.10 | 0.14 | 0.57 | 0.32, 1.02 | 0.061 | 0.96 | 0.56, 1.62 | 0.9 |
| Atrial fibrillation | 332 | 0.99 | 0.67, 1.47 | >0.9 | 0.80 | 0.45, 1.42 | 0.4 | 1.14 | 0.66, 1.98 | 0.6 |
| Previous HF episodes | 332 | 2.00 | 1.27, 3.15 | **0.002** | 3.65 | 1.55, 8.60 | **<0.001** | 1.45 | 0.83, 2.52 | 0.2 |
| Valvular surgery | 332 | 2.00 | 1.22, 3.29 | **0.011** | 1.84 | 0.86, 3.94 | 0.14 | 2.20 | 1.14, 4.24 | **0.031** |
| Pacemaker | 332 | 1.39 | 0.79, 2.43 | 0.3 | 0.49 | 0.12, 2.01 | 0.3 | 1.94 | 1.03, 3.66 | 0.055 |
| Atherosclerotic disease | 332 | 1.56 | 1.06, 2.28 | **0.023** | 1.69 | 0.95, 3.01 | 0.075 | 1.37 | 0.82, 2.30 | 0.2 |
| COPD | 332 | 1.45 | 0.83, 2.55 | 0.2 | 1.51 | 0.64, 3.57 | 0.4 | 1.40 | 0.67, 2.96 | 0.4 |
| Sleep apnoea syndrome | 328 | 0.78 | 0.43, 1.43 | 0.4 | 0.96 | 0.45, 2.07 | >0.9 | 0.61 | 0.22, 1.70 | 0.3 |
| CKD (eGFR <60mL/min) | 332 | 2.09 | 1.30, 3.35 | **0.001** | 2.07 | 1.04, 4.11 | **0.029** | 1.96 | 1.02, 3.78 | **0.032** |
| Diuretics | 332 | 1.49 | 0.90, 2.48 | 0.11 | 2.33 | 0.99, 5.48 | **0.033** | 1.04 | 0.55, 1.97 | 0.9 |
| Spironolactone | 332 | 1.04 | 0.66, 1.64 | 0.9 | 1.42 | 0.74, 2.72 | 0.3 | 0.80 | 0.42, 1.54 | 0.5 |
| Beta-blockers | 332 | 0.75 | 0.51, 1.11 | 0.2 | 0.80 | 0.42, 1.51 | 0.5 | 0.81 | 0.49, 1.34 | 0.4 |
| ACE inhibitors/ARB | 332 | 0.80 | 0.54, 1.18 | 0.3 | 0.88 | 0.48, 1.59 | 0.7 | 0.75 | 0.45, 1.26 | 0.3 |
| Calcium channel blockers | 332 | 1.07 | 0.73, 1.58 | 0.7 | 1.19 | 0.66, 2.17 | 0.6 | 0.91 | 0.55, 1.51 | 0.7 |
| Antiplatelet agents | 332 | 1.38 | 0.94, 2.05 | 0.11 | 1.83 | 1.03, 3.27 | **0.046** | 1.08 | 0.63, 1.84 | 0.8 |
| Oral anticoagulants | 332 | 1.27 | 0.86, 1.89 | 0.2 | 0.95 | 0.54, 1.69 | 0.9 | 1.56 | 0.89, 2.75 | 0.11 |
| Statins | 329 | 0.59 | 0.40, 0.87 | **0.007** | 0.67 | 0.38, 1.19 | 0.2 | 0.55 | 0.32, 0.96 | **0.031** |
| CRP (mg/L) (per SD) | 269 | 1.26 | 1.06, 1.50 | **0.023** | 1.36 | 1.12, 1.64 | **0.017** | 1.11 | 0.82, 1.50 | 0.5 |
| eGFR (mL/min/1.73m²) (per SD) | 331 | 0.61 | 0.48, 0.76 | **<0.001** | 0.60 | 0.43, 0.83 | **0.002** | 0.64 | 0.46, 0.88 | **0.003** |
| Urea (mmol/L) (per SD) | 326 | 1.56 | 1.33, 1.85 | **<0.001** | 1.56 | 1.21, 2.02 | **0.003** | 1.54 | 1.24, 1.92 | **<0.001** |
| Sodium (mmol/L) (per SD) | 325 | 1.03 | 0.84, 1.27 | 0.8 | 0.92 | 0.68, 1.25 | 0.6 | 1.13 | 0.86, 1.50 | 0.4 |
| Haemoglobin (g/dL) (per SD) | 330 | 0.72 | 0.59, 0.87 | **<0.001** | 0.74 | 0.56, 0.97 | **0.029** | 0.70 | 0.54, 0.92 | **0.010** |
| NT-proBNP (pg/mL) (per SD) | 318 | 1.26 | 1.14, 1.40 | **<0.001** | 3.11 | 1.85, 5.24 | **<0.001** | 1.20 | 1.06, 1.35 | **0.012** |
| Troponin (ng/mL) (per SD) | 280 | 1.40 | 1.24, 1.57 | **<0.001** | 1.32 | 1.07, 1.64 | **0.030** | 1.41 | 1.22, 1.63 | **<0.001** |
| Total cholesterol (mg/dL) (per SD) | 296 | 0.83 | 0.68, 1.03 | 0.084 | 0.78 | 0.57, 1.09 | 0.13 | 0.86 | 0.65, 1.14 | 0.3 |
| LDL cholesterol (mg/dL) (per SD) | 294 | 0.97 | 0.79, 1.18 | 0.7 | 0.99 | 0.74, 1.31 | >0.9 | 0.92 | 0.69, 1.22 | 0.5 |
| HDL cholesterol (mg/dL) (per SD) | 295 | 0.67 | 0.53, 0.86 | **<0.001** | 0.71 | 0.50, 1.01 | **0.040** | 0.64 | 0.46, 0.89 | **0.006** |
| Triglycerides (mg/dL) (per SD) | 293 | 0.90 | 0.69, 1.18 | 0.4 | 0.77 | 0.49, 1.20 | 0.2 | 1.08 | 0.75, 1.56 | 0.7 |
| White blood cells (x1,000/mm³) (per SD) | 306 | 1.21 | 1.06, 1.38 | **0.042** | 0.80 | 0.37, 1.74 | 0.5 | 1.20 | 1.06, 1.36 | **0.034** |
| Neutrophile to lymphocyte ratio (per SD) | 306 | 1.25 | 1.06, 1.47 | **0.015** | 1.22 | 0.94, 1.59 | 0.2 | 1.23 | 0.99, 1.54 | 0.078 |
| LA volume Simpsonᵢ (mL) (per SD) | 330 | 1.22 | 1.04, 1.43 | **0.025** | 1.07 | 0.81, 1.40 | 0.6 | 1.30 | 1.05, 1.60 | **0.026** |
| LVEDV Simpsonᵢ (mL) (per SD) | 327 | 1.16 | 0.96, 1.39 | 0.13 | 1.11 | 0.81, 1.50 | 0.5 | 1.18 | 0.94, 1.48 | 0.2 |
| LVESV Simpsonᵢ (mL) (per SD) | 327 | 1.12 | 0.94, 1.34 | 0.2 | 1.14 | 0.86, 1.51 | 0.4 | 1.11 | 0.89, 1.38 | 0.4 |
| LVEF Simpsonᵢ (per SD) | 332 | 0.92 | 0.76, 1.11 | 0.4 | 0.81 | 0.59, 1.11 | 0.2 | 0.97 | 0.76, 1.22 | 0.8 |
| SV (mL) (per SD) | 310 | 0.77 | 0.63, 0.96 | **0.014** | 0.75 | 0.53, 1.05 | 0.082 | 0.80 | 0.61, 1.04 | 0.086 |
| Mitral E (cm/s) (per SD) | 328 | 1.29 | 1.08, 1.55 | **0.007** | 1.05 | 0.79, 1.40 | 0.7 | 1.60 | 1.26, 2.04 | **<0.001** |
| Septal E' (m/s) (per SD) | 326 | 0.87 | 0.70, 1.08 | 0.2 | 0.92 | 0.67, 1.27 | 0.6 | 0.88 | 0.65, 1.19 | 0.4 |
| E/e' septal (per SD) | 326 | 1.36 | 1.15, 1.60 | **<0.001** | 1.18 | 0.89, 1.56 | 0.3 | 1.45 | 1.19, 1.77 | **<0.001** |
| Tricuspid insufficiency | 329 | 1.81 | 1.14, 2.88 | **0.018** | 1.68 | 0.75, 3.76 | 0.2 | 1.74 | 0.98, 3.10 | 0.070 |
| RV FAC (%) (per SD) | 298 | 0.80 | 0.65, 1.0 | **0.044** | 0.53 | 0.37, 0.77 | **<0.001** | 1.02 | 0.77, 1.33 | >0.9 |
| TAPSE (mm) (per SD) | 296 | 0.70 | 0.56, 0.86 | **<0.001** | 0.63 | 0.47, 0.85 | **0.002** | 0.78 | 0.58, 1.05 | 0.091 |
| Tricuspid regurgitation velocity (m/s) (per SD) | 330 | 1.37 | 1.08, 1.73 | **0.006** | 1.26 | 0.91, 1.75 | 0.14 | 1.42 | 1.01, 2.00 | **0.040** |
| ePAP (mmHg) (per SD) | 319 | 1.40 | 1.15, 1.71 | **0.001** | 1.40 | 1.04, 1.88 | **0.032** | 1.34 | 1.01, 1.78 | **0.045** |
| TAPSE/ePAP (mm/mmHg) (per SD) | 284 | 0.54 | 0.34, 0.86 | **0.001** | 0.69 | 0.42, 1.13 | 0.072 | 0.40 | 0.19, 0.87 | **0.012** |
|  | | | | | | | | | | |

Abbreviations: ACE: angiotensin-converting enzyme; ARB: angiotensin receptor blocker; BMI: body mass index; CAD: coronary artery disease; CKD-EPI: chronic kidney disease - epidemiology collaboration; CI: confidence interval; COPD: chronic obstructive pulmonary disease; CRP: C-reactive protein; eGFR: estimated glomerular rate; ePAP: estimated pulmonary artery systolic pressure; HDL: hidh-density lipoprotein; HF: heart failure; HR: hazard ratio; LA: left atrial; LDL: low-density lipoprotein; LVEDV: left ventricular end‐diastolic chamber volume; LVEF: left ventricular ejection fraction; LVESV: left ventricular end‐systolic chamber volume; NT-proBNP: N-terminal pro b-type natriuretic peptide; NYHA: New York heart association; RV FAC: right ventricular fractional area change; SD: standard deviation; SV: stroke volume; TAPSE: tricuspid annular plane systolic excursion.

**Figure S1.** Bivariable Cox regression restricted cubic spline for age and all-cause mortality. Reference set at 60 years.

**Figure S2.** Age-specific death risk of the Belgian population.
